# Engineering Features from Raw Sensor Data to Analyse Player Movements during Competition

**DOI:** 10.3390/s24041308

**Published:** 2024-02-18

**Authors:** Valerio Antonini, Alessandra Mileo, Mark Roantree

**Affiliations:** 1 School of Computing, Dublin City University, Dublin 9, D09 V209 Dublin, Ireland; alessandra.mileo@dcu.ie (A.M.); mark.roantree@dcu.ie (M.R.); 2 Insight Centre for Data Analytics, School of Computing, Dublin City University, Dublin 9, D09 V209 Dublin, Ireland

**Keywords:** feature engineering, wearable devices, machine learning

## Abstract

Research in field sports often involves analysis of running performance profiles of players during competitive games with individual, per-position, and time-related descriptive statistics. Data are acquired through wearable technologies, which generally capture simple data points, which in the case of many team-based sports are times, latitudes, and longitudes. While the data capture is simple and in relatively high volumes, the raw data are unsuited to any form of analysis or machine learning functions. The main goal of this research is to develop a multistep feature engineering framework that delivers the transformation of sequential data into feature sets more suited to machine learning applications.

## 1. Introduction

Technological advancements have made it possible to measure positions, motion, and inertial forces related to human movements with several types of new instruments within the field of wearable sensors. Due to their low cost, cutting-edge technology, and easy portability, wearable sensors have been widely used in the last decade to assess and analyse physical activity [1,2,3], game demands [4], and external loads in sports analytics [5,6,7]. The capability of wearable devices for assessing and measuring human physical performances enables them to become a fixture in the near future of sports science.

In the case of GPS technology, these devices transmit the time of orbiting satellites synchronised with GPS receivers on Earth using an atomic clock. By exploiting the signals that the GPS sensor on Earth receives from at least four GPS-orbiting satellites, it is possible to compute the position of the GPS receiver [8]. The validity and reliability of the GPS sensor have been already assessed [9,10,11]. The data collected from the micro wearable devices record the physical load expenditure of athletes during training sessions and games and can be harnessed to assess players’ performances [12], prevent injuries [13,14], quantify match running performances [15], and optimise rehabilitation training [16].

This study uses data collected during games of Gaelic Football (GF), a sport that originated in Ireland, with unique rules that make it a combination between rugby and soccer. Players can carry the ball in their hands but must bounce it to the ground or tap it with their foot every four steps. It is possible to pass the ball with both hands and feet. The goalpost is similar to rugby, and it is possible to score above the crossbar (one point) or below it (three points). GF games are played by two teams of 15 amateur players. The size of the pitch is about 130–150 m in length and 80–90 m in width. GF can be played at the club (local) or county (regional) levels. County games last 70 min, with two halves of 35 min each.

### 1.1. Problem Statement and Contribution

Studies on Gaelic Football aim to furnish comprehensive running performance profiles for players engaged in competitive matches. This is enabled by wearable technologies, which automate the capture of high volumes of information during most or all sporting activities, such as training and matches. Despite the fact that this form of data harvesting has been widespread for over a decade, the application of machine learning and deep learning techniques is still in the embryonic stage. More complex analyses, such as the prediction of players’ running performances, is not currently addressed. To date, player running profiles have primarily been established through retrospective analysis of historical data. Recent advancements in machine learning techniques have unlocked the potential for forecasting forthcoming events and generating insights. However, automated data acquisition as typically offered by GPS devices delivers data purely on the basis of the location at a specific time, albeit at a very granular level. While some manufacturers will offer to transform this into a more usable format (effort in terms of speed and distance), it is not necessarily at a level suited to machine learning functions and, in some cases, suffers from information loss due to the highly abstract nature of the report data [17].

In this research, we present a multistep framework to manipulate raw sensor values with the objective of creating a feature set suited to predicting further actions or events during game time analysis. In effect, it offers a more data-centric approach to decision making in sports coaching and management. As part of this process, we articulate the contribution of this work as follows:

- A methodology with a parameterisable ruleset for feature extraction from raw sensor data. In effect, a per-second stream of locations is converted to a sequence of actions.
- An algorithm to convert a sequence of actions into an event where the event captures movement from (close to) rest and a return to the same resting state.
- The application of existing research, covering typical in-game player workloads, to offer a comparative validation for our feature set creation. As part of this validation, a dimensional analysis of game/player workloads is presented by game (time), player, or action.
- Finally, as a proof of concept for the application of machine learning to predict player performances towards the end of the game, the first half of a selected game plus the other previous games are used to predict the number of metres covered at high speed for the second half, with a discussion as to how these machine learning functions performed over the task.

### 1.2. Paper Structure

Section 2 provides a discussion of past feature engineering methods on sensor data and past GF research on players’ running performances. Section 3 describes the data acquisition and the multistep framework to obtain the Actions and Events datasets. Section 4.2 presents the validation of the Actions dataset, a set of statistics on the players’ actions and events during gameplay, and the prediction of forthcoming events. Concluding remarks are presented in Section 6.

## 2. Related Research

### 2.1. Feature Engineering

Data collected by wearable sensors represent a time series comprising latitude, longitude, and speed values. If properly analysed and processed, it is possible to extract valuable information and insights [18]. Time series can be analysed directly, or it is possible to extract features describing trends and variances [19]. A valid and effectual method for pattern recognition in time series analysis is to define the time series with respect to the distribution of data points, correlation properties, trends, and data spread [19]. The engineering of the raw data for obtaining new transformed features is a common process in GPS data analytics. The researchers in [20] proposed a transportation-mode classification method based on combining feature engineering techniques and a Light Gradient Boosting Machine to discover seven kinds of transportation modes from GPS trajectory data. The original trajectories were divided into sub-trajectories (sequences of GPS data points), where each sub-trajectory represents a unique transportation mode. Then, on these sub-trajectories were extracted the following features: the distance, average velocity, expectation velocity, expectation acceleration, 95th percentile velocity, 95th percentile acceleration, minimum velocity, variance velocity, heading change rate, stop rate, and velocity change rate. Working on the same dataset, the authors in [21] used a different set of features for the classification. In addition to the speed and acceleration, they computed the jerk, bearing, and bearing rate for each data point. Therefore, for each sub-trajectory example, they extracted the minimum, maximum, mean, median, standard deviation, and 10th, 25th, 50th, 75th, and 90th percentile for each of the specific features.

A follow-up study [22] improved the prediction results by adding additional features, both statistical attributes (kurtosis, skewness, coefficient of variation, and autocorrelation coefficient) and domain knowledge (stop rate, velocity change rate, and head change rate). The new set of statistical features benefits the final prediction [22], but some of them are slightly different measures even for the same concept (for example, the standard deviation and coefficient of variation have a similar formula and record the spread of data). This can lead to, for example, multicollinearity (an approximately linear relationship between two or more independent variables) in the data, which can damage the effectiveness of the model [23].

To recognise modes of driving railway trains from GPS data, the authors in [24] derived multiple features from the speed of locomotives during routes (in addition to some domain-specific features). The set of features can be grouped into global statistical features (mean, standard deviation, mode, median, max three consecutive values, min three consecutive values, value range, interquantile range, skewness, kurtosis, coefficient of variation, autocorrelation coefficient, stop rate, velocity change rate, distance), local statistical features (mean length of each decomposition class, length standard deviation of each decomposition class, proportion of each decomposition class, change times), and time-domain and frequency-domain features (median crossover rate, number of peaks, short-time Fourier transformation).

### 2.2. Players’ Running Performance Research in Gaelic Football

In the last decade, sports institutions and teams developed a deep interest in data-driven approaches, aiming to support decision makers in obtaining a competitive advantage. Data obtained from wearable sensors can be exploited in several ways, from describing, planning, and monitoring external loads to injury prediction. External loads in invasion field-based team sports (IFTSs) can be described by measures of total distance covered (or in specific speed bands), accelerations, or metabolic power [25]. Such metrics are the result of defined computation on data obtained via tracking systems (wearable devices) worn by the players during training sessions or official games.

The authors in [15] analysed the match running profiles of GF players. They collected data from 50 male GF players using a 4 Hz GPS sensor across 30 competitive games with a total of 212 full-game datasets collected. The variables analysed were the following: high-speed distance, sprint distance, mean velocity, peak velocity, and number of accelerations. The average match distance covered by the players was 8160 ± 1482 meters m with 1731 ± 659 m covered at high speed. The sprint distance was 445 ± 169 m with 44 sprint actions on average per match. The peak velocity was 8.4 ± 0.5 m/s with a mean velocity of 1.8 ± 0.3 m/s. The number of average accelerations completed per player was 184 ± 40. Significant differences between positions were found for total running distance, high-speed running distance, and sprint distance, with midfielder being the position most demanding for the variables total and high-speed running distance. The researchers found a significant reduction in the high-speed and sprint distance between the first and second half.

In later work by the same authors [26], they aimed to identify the position and duration of the running performance of GF players using a moving average window. The study was performed on 35 players across 32 competitive matches analysed with 300 full-match play data samples using a 4 Hz GPS sensor. Players were grouped based on their position: full-back, half-back, midfield, half-forward, and full-forward. The researchers tested ten different lengths (1 to 10 min) for the moving average to analyse the recorded speeds.

The researchers in [27] quantified the running profiles of GF players according to their position and evaluated the trend of physical performance during the games. The average relative distance recorded was 92.4 ± 23.3 m per minute m/min, composed of 28.4 ± 10.2 m/min covered at a speed ≥ 4 m/s (high-intensity running) and 9.9 ± 3.9 m/min at a speed ≥ 5.5 m/s (very high intensity running). The distance covered at high-intensity running was higher in half-backs, midfielders, and half-forwards compared to the full-backs and full-forwards. The time window P1, grouping the first 15 min of the first half (0–15 min), observed the highest amount of distance covered at each speed zones, with a linear decrease during the windows P3 (40–55, first 15 min of the second half), P2 (15–30), and P4 (55–70).

The differences in running performance between GF positions have been investigated by [28]. The running performance is dependent on the position on the pitch (p<0.001). Midfielders cover a greater amount of total and relative distances compared to the other positions. Half-backs and half-forwards ran greater total distances compared to full-forward and full-backs. Midfielders were also observed to cover a greater amount of distance at high speeds compared to all other positions, while half-backs and half-forwards traversed distances at high speeds more than the other remaining position groups. Sprinting distances were covered more by half-forwards, followed by half-backs and midfielders.

Similar differences in running performance between different GF playing positions have been confirmed in [29]. The authors found that the midfielders covered a significantly greater distance than defenders and that the frequency of jogging, cruise running, striding, and walking was greater in midfielders rather than in the forward positions [29].

The study [30] reported that GF players perform 166 ± 41 accelerations per game. The high-speed running distance and very high speed running distance were reported to be equal to 1563 ± 605 and 524 ± 190 m, respectively. The average distance covered during acceleration is 267 ± 45 m, distributed at 12 ± 5 accelerations per 5 m epoch. The maximum distance covered in acceleration is 296 ± 134 m.

### 2.3. The Concept of Movement Event in Sport Science Research

The concept of the movement event used in this research is based on the study [31], which designed a new approach to detect recurrent movements in sports by analysing positional data. Speed, acceleration, and angular velocity values were obtained from sequential sequences of positional data and clustered with the *k*-means algorithm, in order to create subgroups of values. Each action is composed of three values for the three dimensions. Sequences of actions were compared, assessing similarities using the longest common sub-string algorithm [31].

The authors in [32] proposed a framework for extracting movements from sequential GPS data. Similar to [31,32], they combined the speed, acceleration, and direction of the movement (turning angle) for defining a player movement description, utilising thresholds for each movement descriptor in order to assign individual labels (letters). Each sequence of movements starts and ends when a player is at a speed less than or equal to 1.2 m/s. The researchers in [32] compute distances among movement descriptors using the Levenshtein distance, which computes the number of required steps to transform one list of strings into another.

Based on the concept of an event as a sequence of actions between resting states defined in [31,32], this research satisfies the necessity of identifying profiles of events. Events are analysed using frequencies, the number of actions, the average and max duration, and the average and max distance covered.

**Summary.** Table 1 summarizes the key insights and contributions from past research relevant to our study.

We build on each of the three areas in the research described above, as we have developed a novel multistep framework, designed for the purpose of feature engineering raw sensor data into multi-granular datasets, for the purpose of exploiting machine learning functions to predict future events during game time. To the best of the authors’ knowledge, this framework represents a pioneering approach directed towards the acquisition and manipulation of features, to enable both machine learning applications and the analysis of actions and events utilising sensor data within the field of sport analytics. In addition, predictive modelling techniques are employed to forecast the high-speed distance of forthcoming events during the second half of competitive games based on the acquired data from the first half and the previous games.

## 3. Enrichment of Raw Sensor Data

**Figure 1:**
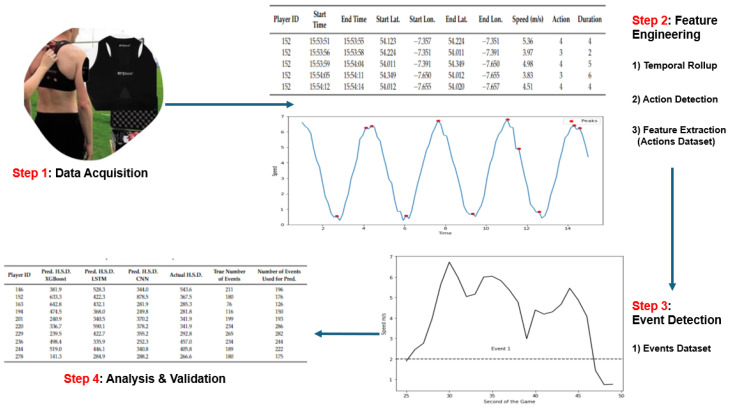
Transformation methodology.

In this section, we present a multistep method illustrated in Figure 1, which transforms raw sensor data into a series of higher-level events, which are more easily analysable. Our method is based on the concepts of actions and events, which deliver data more suited to rich forms of analysis and a broad range of machine learning functions. In summary, for each second of the game, speeds are converted to one of six action labels: ‘standing’, ‘walking’, ‘jogging’, ‘running’, ‘high-intensity running’, and ‘sprinting’, according to velocity thresholds widely accepted and defined in the literature [34]. An event is considered to be a collection of sequential actions, bookended by either ‘standing’ or ‘walking’ actions. In summary, players are regarded as being relatively static before commencing into some form of movement or sequence of actions. This feature engineering process is underpinned by a set of rules or logic taken from domain experts and the existing literature. The values shown in Table 2 represent six activity zones, which are standard across many sports, making this feature extraction process widely generic. Furthermore, the adjustment of the parameters or values within zones enables a customisation across sports, gender, and age.

### 3.1. Data Acquisition

The first step shown in Figure 1 is data acquisition, which refers to the generation of data on devices strapped to players and the extraction of that data from each device.

For this study, data were collected during 11 competitive GF inter-county games throughout the seasons 2019–2021. The players wore a micro GPS sensor device (STATSports Apex 10 Hz, Northern Ireland, UK), placed on the upper back, within a vest that fitted the body comfortably. Previous research assessed the accuracy and reliability of the specific sensor involved [11]. The study claims that the 10 Hz Apex unit reported a small error of around 1–2% of the distances measured in the experiments and that the unit could be used with confidence to measure distance variables during training and match play [11].

The GPS sensor records 10 observations for each second for the variables latitude, longitude, and speed (m/s). The raw data, shown in Table 3, were collected only when the athletes were actively taking part in the game and were provided already filtered by the STATSports software (version: 4.5.19).

### 3.2. Aggregation (Temporal Rollup)

The Data Aggregation step (which we refer to as temporal rollup) is necessary as data are sampled at too fine a level of granularity for our research purposes. Raw data files may, for example, contain data sampled at 10 Hz (10 observations per second). For the scope of this paper, a single observation per second provides a sufficient level of detail, which, in effect, is a temporal rollup to 1 s values. Thus, the input to this step is the 10 Hz dataset and the output is a dataset with one observation per second.

Our decision was to adopt the average values within each 1 s interval, as it provides a good approximation for describing movement within that timeframe. The location (latitude and longitude) is transformed to the centroid of the locations covered, while the variable speed would be the average values during the second (Table 4).

### 3.3. Feature Extraction

The 1 s interval data now serve as input to this second step where the goal is to further aggregate the data: in this case, a rollup to the level of action. Each data point will no longer represent 1 s of activity but an entire action, which will likely run over a number of seconds. This is carried out using a set of speed thresholds demarcating the transition from one movement type to another.

#### 3.3.1. Parameter Definition

No standardised set of speed thresholds is available for IFTSs [35] to classify a player’s movement. Speed thresholds suggested by [34], which are commonly used in IFTSs, were therefore adopted. Each speed value $x_{i}$ (i=1,2,…,n, where n is the last second of the game) recorded in the instant of time *i* is converted to an action using the labelled thresholds (Table 2).

In addition, the acceleration is computed by subtracting each speed value from the previous one. A sample of the dataset after this step is shown in Table 5.

Algorithm 1 illustrates the DetectAction algorithm. This algorithm sequentially processes the rows of a dataset related to one of the halves of a game played by a single player. It effectively compresses similar actions into a unified row, simplifying a sequence of consecutive actions. It computes various features on the identified actions, such as the minimum and maximum values, the absolute energy (sum of squared values), the count of values above the average, the sum of all values (sum over the time series values), the length of the longest consecutive subsequence above the mean (longest strike), and the coefficient of variation ($\frac{\mathrm{standard}\;\mathrm{deviation}}{\mathrm{mean}}$) for the variables speed and acceleration. Additionally, the number of accelerations and decelerations are measured as the count of times the acceleration values were ≥2 and ≤−2.
**Algorithm 1** DetectAction algorithm**Require:** *N* rows of dataset
**Require:** N≥2
**Require:** $x_{z}$ action performed by the player at iteration *z* **Require:** $t_{z}$ time of the game at iteration *z*
1:avg_speed←02:avg_speed_list← []3:start_time_list← []4:end_time_list← []5:y←current_action6:z←07:**while** i≤N **do**8:      **if** $x_{i}=y$ **then**9:         continue10:    **else if** $x_{i}\neq y$ **then**11:        $avg\text{\_}speed\leftarrow avg(x_{z},\ldots ,x_{i-1})$12:        avg_speed_list[z]←avg_speed13:        $start\text{\_}time\text{\_}list\left[z\right]\leftarrow t_{z}$14:        $end\text{\_}time\text{\_}list\left[z\right]\leftarrow t_{i-1}$15:        z←z+116:        y←x_i17:    **else if** i=N **then**18:        avg_speed←avg(x_z,…,x_i)19:        avg_speed_list[z]←avg_speed20:        $start\text{\_}time\text{\_}list\left[z\right]\leftarrow t_{z}$21:        $end\text{\_}time\text{\_}list\left[z\right]\leftarrow t_{i}$22:    **end if**23:**end while**

The algorithm also captures the time when the action occurred, the start and end locations on the pitch, and the duration of the action in seconds. It is important to mention that actions are recorded with a minimum duration set at 1 s.

Algorithm 1 exclusively displays the variables of average speed, starting time, and ending time. The variables recording the starting and ending locations adhere to the rule of the starting and ending times, while the remaining variables (min, max, absolute energy, longest strike, etc., both for speed and acceleration) follow the criterion of average speed.

Algorithm 1 iterates through the N rows of a dataset (Table 5) comprising the sequence of the time, location, speed, action, and acceleration of a player during a game. At each iteration *i*, the action performed by a player is indicated by the variable $x_{i}$ and the time when the action occurs by the variable $z_{i}$.

The first step of the algorithm is to initialise the required variables avg_speed, avg_speed_list, start_time_list, end_time_list, y, and z, which will measure the average speed during the action, the list of such speeds, the time when the action begins and the time when it ends, the current stored action, and the row when the action starts, respectively (1–6). While iterating over each row of the dataset (which has a length equal to N) (7), if the current performed action is equal to the current stored action, then it continues with the next row (8, 9). If the two actions are different (10), then the average speed from the row when the action starts to the current row is computed (11) and stored in the list of the speeds (12), the starting time and ending time of the action are added to the corresponding lists (13, 14), and the variables storing the row when the action starts (z) and the current action (y) are updated (15, 16). If the row is the last row of the dataset (17), the algorithm computes the average speed until this row (18), it stores the value in the list of the speeds (19), and it adds the starting time and ending time of the action to the corresponding lists (20, 21).

Algorithm 1 requires the data points to be consecutive on a per-second basis. It thus works on one half per time. It is suggested to apply it individually to each half of the game for each player and subsequently concatenate the results.

A sample of the resulting dataset after this step is shown in Table 6 (some of the features introduced in this section have been omitted).

#### 3.3.2. Direction of Movement

In order to detect the direction of movement, the next step is the computation of the turning angle from the position at time *i* (beginning of the action) and the position at i+1 (end of the action) [32] (i=1,2,…,n, where *n* is the last second of the game). The turning angle is the angle you would need to turn from the starting direction to the ending direction in order to reach the destination. For obtaining the turning angle, the bearing for the two consecutive points should be computed and then subtracted. The bearing is used to describe the direction or angle between two points, measured clockwise from the magnetic north. It represents the direction you would need to travel in a straight line from point A to point B, measured in degrees. There are different steps needed for computing the bearing:

1. Compute *X* and *Y* [32]:
  (1) $$\begin{array}{c} X=\cos(\theta B)\sin(\Delta L), \end{array}$$
  (2) $$\begin{array}{c} Y=\cos(\theta A)\sin(\theta B)-\sin(\theta A)\cos(\theta B)\cos(\Delta L), \end{array}$$
  where θ denotes the latitude, L denotes the longitude, *A* and *B* denote two consecutive decimal coordinates, and ΔL denotes the difference between L at A and L at B.
2. Compute the bearing β [32]:
  (3) $$\begin{array}{c} \beta =\arctan2(X,Y), \end{array}$$
3. The turning angle τ is obtained by subtracting the bearing computed on two successive time points [32]:
  (4) $$\begin{array}{c} \tau =\beta_{i}-\beta_{i-1}, \end{array}$$

The numerical turning angle values are expressed as degrees from $0^{\circ }$ to $360^{\circ }$ and are then converted to a label (forward, backward, right, and left) expressing the direction of movement (Table 7)

### 3.4. Event Detection

**Figure 2:**
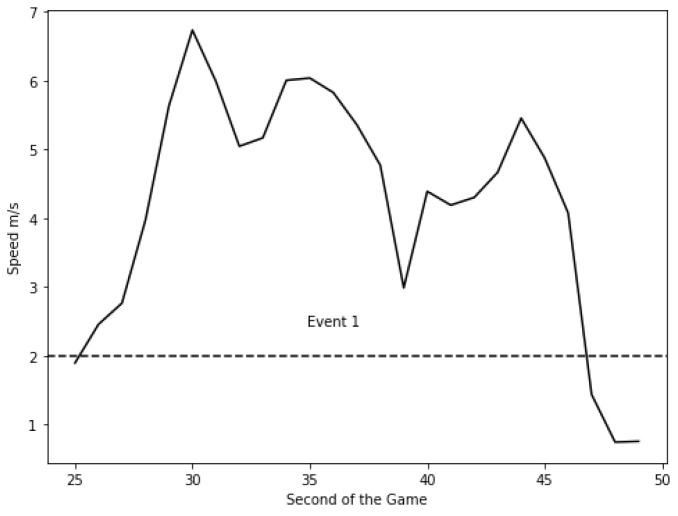
Event identification from the raw sensor data.

As per our earlier definition in Section 3.2, an event is a sequence of actions that occur between two resting states ‘standing’ or ‘walking’. Therefore, an event commences when the player transitions out of a ‘standing’ or ‘walking’ state (speed ≤2 m/s) to performing actions at higher speeds ‘jogging’, ‘running’, etc. and finally returns to a ‘standing’ or ‘walking’ movement (Figure 2). Each event can be of different lengths and compositions but all start and end with ‘standing’ or ‘walking’ unless some unexpected interruption occurs. This definition of an event is based on past research [31,32].

The DetectEvent algorithm (Algorithm 2) iterates through the N rows of the dataset corresponding to one half of a player’s game (Table 6) to identify actions associated with the same event.
**Algorithm 2** DetectEvent algorithm**Require:** N≥2 **Require:** 
$x_{z}$ action performed by the player at the iteration *z*
1:$y_{s}\leftarrow$ ‘*Standing*’2:$y_{w}\leftarrow$ ‘*Walking*’3:events_list← []4:id_event←05:current_state←06:**while** i≤N **do**7:    **if** $x_{i}=y_{s}$ or $x_{i}=y_{w}$ **then**8:        **if** current_state=1 **then**9:             id_event←id_event+110:           events_list[i]←id_event11:        **end if** current_state=012:        events_list[i]←id_event13:        current_state←114:    **end if** $x_{i}\neq y_{s}$ and $x_{i}\neq y_{w}$15:    events_list[i]←id_event16:    current_state←017:**end while**


Algorithm 2 begins with the initialisation of variables: $y_{s}$, $y_{w}$, events_list, id_event, and current_state, representing a variable equal to ‘standing’, a variable equal to ‘walking’, the list of the events ids, the current event id, and the current state, respectively (1–5). Current state is an indicator that contains 0 the first time the algorithm meets an action equal to ‘standing’ or ‘walking’ or 1 if it has already met one. While iterating over the dataset (6), if the current action x_i is equal to ‘standing’ or ‘walking’ (7), two situations can occur. If the current state is equal to 1 (8), a new event starts: the id_event is increased by 1 (9), and the new value is added to the list of events (10). On the contrary, if the current state is equal to 0 (11)), add the id_event to the list of the events (12) and switch the value of the variable current state to 1 (13). If the current action x_i is different from ‘standing’ or ‘walking’ (14), add the current id_event to the list of the events (15) and set the current state to 0 (16).

At the end of this step, each action is assigned to an event with a unique identifying label (Table 8).

The final step in constructing the Events dataset involves the aggregation of actions and action features into events. The Actions dataset is aggregated into the Events dataset by grouping rows sharing the same event ID. Each row of the final Events dataset represents an event performed by a player in a game.

Table 9 displays a sample of the resulting Events dataset. ‘player ID’ indicates the ID of the player; the ‘event ID’ indicates the ID of the event; ‘start_second’ and ‘end_second’ indicate the starting and ending seconds from the beginning to the game ‘start_time’ and ‘end_time’, expressed in Table 6 as a specific time in the format HH:MM:SS, have here been converted); ‘start_lat’, ‘start_lon’, ‘end_lat’, and ‘end_lon’ indicate the starting and ending locations of the event; ‘avg speed’ and ‘std speed’ indicate the average and the standard deviation of the speed of the actions composing the event; ‘duration’ indicates the duration of the event; ‘std duration’ indicates the standard deviation of the duration of the actions composing the event; ‘distance’ indicates the distance covered during the event; high-speed distance’ indicates the distance covered at speed ≥5.5 m/s during the event; and ‘unique turning angles’ indicate the number of different directions of movement during the event.

The average and standard deviation has been calculated for the statistical features derived from the speed and acceleration values of each action and presented in Section 3.3.1. These features include the minimum and maximum values, absolute energy, count of values above the average, sum of all values, length of the longest strike, and coefficient of variation. Furthermore, the total count of accelerations and decelerations has been calculated for each event.

Not all the collected features have been displayed in Table 9 due to space reasons.

## 4. Statistical Summaries and Feature Set Validation

To validate the framework, metrics previously adopted in GF research are applied to the Actions dataset, aiming to assess whether findings in this research align with those from prior studies. Then, a statistical analysis of the data comprising the constructed dataset is provided. Speeds, distances, actions, and events are analysed by halves or matches in order to show trends, patterns, similarities, and differences.

Several statistical *t*-tests on the variables have been conducted to investigate differences between the first and second halves of games. Finally, three predictive models are tested on the Events dataset to forecast the high-speed distance covered by players during the second half of a selected game, based on the first half and the remaining games.

### 4.1. Actions Dataset Evaluation

In order to verify that the Actions dataset is a true reflection of the actual game, it would be necessary to watch and record the movements of all 15 players in real time, a process that is both impractical and would not scale. Instead, our decision was to compare the dataset with the existing literature to ensure that our feature engineering process delivers a dataset that is in line with existing analyses in terms of the overall volumes and intensity of games. In this respect, we investigated if running profiles are coherent with previous findings in GF research using the in-game profile of elite male Gaelic Football in [15]. The authors identified an average distance covered of 8160.11 ± 1482.02 m, with 1731.29 ± 659.76 m covered at speed 4.72 m/s and 445 ± 169 m at speed 6.11 m/s. Similar values are observed in the Actions dataset: the average distance identified is 8633.8 ± 1573.6 m, with 1453.6 ± 552.7 m covered at high speeds of 4.72 m/s and 503.5 ± 205.1 m at a sprinting speed of 6.11 m/s.

Consistent outcomes were observed even for both the average and peak speeds: 1.8 ± 0.3 m/s and 8.4 ± 0.5 m/s, respectively, in [15] and 2.8 ± 1.6 m/s and 8.4 ± 0.5 m/s in this current research.

In a separate study conducted using the same data [36], the authors observed a reduction in the distance covered between the first and other quarters of the game: −4.1% with the second, −5.9% with the third, and −3.8% with the fourth. They observed a reduction in high-speed running distance (≥4.72 m/s) in the second (−8.8%), third (−15.9%), and fourth (−19.8%) quarters when compared to the first quarter. This research found, in part, a similar reduction in the distance covered between the first and other quarters of the game: −6.6% with the second, −6.6% with the third, and −15.8% with the fourth. When comparing the high-speed running distance between the first and other quarters, the reductions were equal to −7.2% (second), −11.3% (third), and −17.1% (fourth).

The previous comparisons reveal a consistent analogy between this research’s findings with those obtained by prior GF research. The marginal differences identified may be attributed to the different sample sizes or players’ fitness levels.

### 4.2. Games: Half by Half Analysis

The goalkeeper has been omitted from the analysis due to the difference in physical demands. The average speeds during the first and second halves are 2.84 ± 1.62 m/s and 2.73 ± 1.61 m/s, respectively. The *t*-test reports a statistically significant difference between the two means (*p*-value: $1.89\times 10^{-12}$).

The count, average, and standard deviation of the duration and maximum duration of the aggregated actions are shown in Table 10. The mean count of actions for each game is 3945 ± 286. The average duration of the actions is 2.9 ± 0.1 s. The average of the maximum duration of the actions is 25.2 ± 3 s.

The resulting *p*-value for the *t*-test on the count of actions is 0.004 (lower than the chosen level of significance, 0.05), showing a statistically significant difference for the number of actions in the two halves of the game, which is always higher in the first half.

### 4.3. Analysis of Extracted Actions

An analysis of all labelled actions is presented in Table 11. The average number of actions per game decreases as the speed increases: ‘jogging’ (4400.5 ± 226.5), ‘running’ (2402.5 ± 138.9), ‘high-intensity running’ (827.9 ± 54.3), and ‘sprint’ (155.1 ± 14.6).

A *t*-test has indicated the presence of a statistically significant difference in the mean duration of ‘high-intensity running’ actions between the first half (2.0 ± 0.1 s) and the second half (2.2 ± 0.1 s). In reference to the other actions, the mean duration of ‘jogging’, ‘running’ and ‘sprint’ actions are 3.4 ± 0.1, 2.3 ± 0.1, and 2.1 ± 0.2 s, respectively.

A statistically significant difference has been identified through a *t*-test for the mean distance per ‘high-intensity running’ action between the first and second halves (12.4 ± 8.7 and 13.1 ± 9.6 m).

The remaining actions revealed similar outcomes when comparing the two halves.

### 4.4. Analysis of Events

Events composed only of the actions ‘standing’, ‘walking’, and ‘standing’ and ‘walking’ are removed from the current analysis.

The average number of events during the first and second halves are 1657.3 ± 106.7 and 1538.2 ± 94.9, respectively.

The highest number of events during the first half is 1937, with 7.1 ± 6.1 s of average duration and 63 s of maximum duration for an event (Table 12). The highest for the second half is 1669 with 7.1 ± 6.2 s of average duration and 72 s of maximum duration for an event.

The average number of actions per event is 2.55 ± 2.25, with 1 as the minimum and 21 as the maximum.

**Figure 3:**
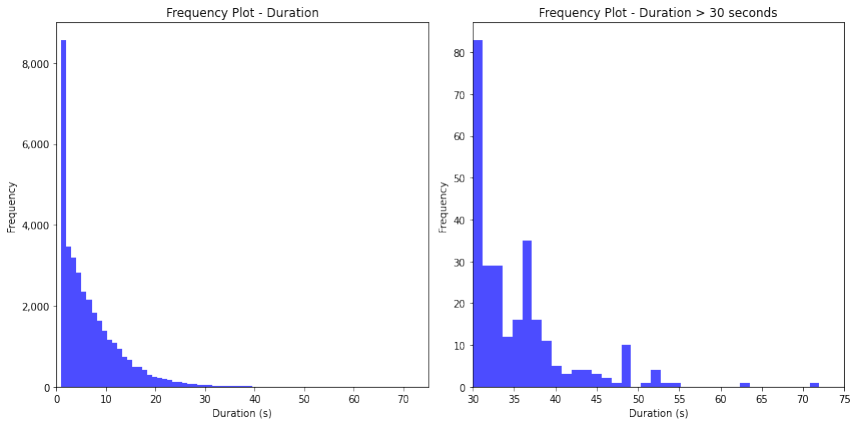
**Left**: Frequency of events per duration. **Right**: Frequency of events per duration ≥ 30 s.

**Figure 4:**
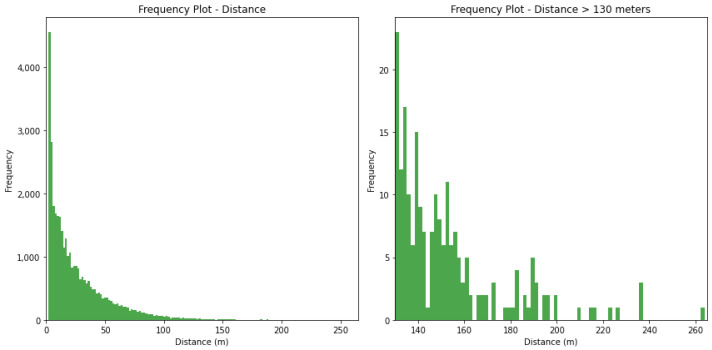
**Left**: Frequency of events per distance. **Right**: Frequency of events per distance ≥ 130 m.

**Figure 5:**
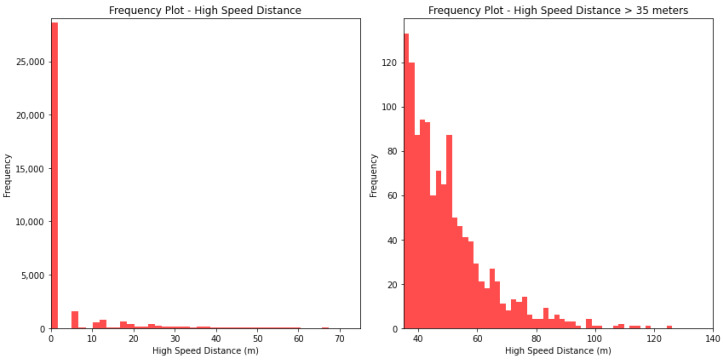
**Left**: Frequency of events per high-speed distance. **Right**: Frequency of events per high-speed distance ≥ 35 m.

Figure 3, Figure 4 and Figure 5 show the frequency of values for the features ‘duration’, ‘distance’, and ‘high-speed distance’. The shape of the frequency distribution is similar for the three investigated features, characterised by elevated frequencies at lower values and a visible decline as values increase.

## 5. Case Study: Predicting High-Speed Actions

While the main focus of this work is on engineering feature sets from the raw sensor data, it is useful to illustrate a case study of the types of possible analyses using the new feature set. Here, we run a number of experiments to predict the high-speed distance covered by players during games, comparing the results obtained by three different machine learning models.

Knowing this metric in advance can be useful for sports coaches to manage players’ time on the pitch, for example, to avoid fatigue or even injuries. The challenge is to predict, for each player, the high-speed distance covered per event during the second half of a target game. Then, these predicted distances are summed to obtain the predicted total high-speed distance per player.

The dataset used to complete this task is a transformation of the Event dataset (Table 9). For each player, a sequence of 30 consecutive high-speed distance values is used as a new set of features, and the variable to predict is the high-speed distance at the 31st position (Table 13).

**Figure 6:**
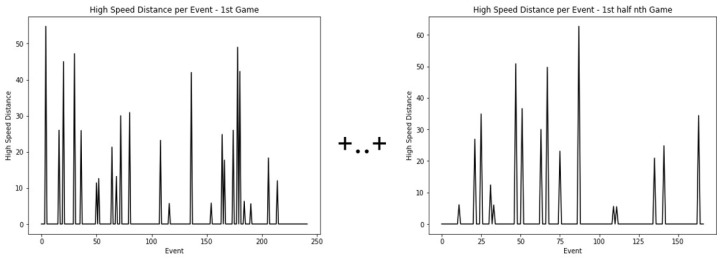
The time series of high-speed distance for the remaining games are merged to form a unique time series. The first half of the tested game is placed in the last position. These data constitute the training set for the predictive experiments.

To replicate a real-world scenario, each model predicts the high-speed distance values for the second half of the last game in a sequence of games, using training using data from the first half of the same game and all prior games in the sequence (in this case study, 10 previous games). The time series data of high-speed distance for the remaining games plus the first half of the tested game are merged to form a unique time series. The first half of the tested game is placed at the end of this time series, as shown in Figure 6. These data constitute the training set for the predictive experiments.

While the learning phase uses data from prior games together with the first half of the target game, during the testing phase, the test set is the model’s predictions. This approach is known as recursive forecasting. In this method, the model is trained on historical data, and rather than having a separate test set, it uses its predictions as inputs for future predictions. This experiment simulates the real scenario of the second half distances being forecast during the half-time break based on data from the first half and the previous games.

Assuming *N* as the number of events performed by player *i* during the first half, validation can thus be described as follows:

1. The sequence composed of the last 30 high-speed distance values of the first half is used as input (test set) by the model to generate the first prediction.
2. The sequence composed of the last 29 high-speed distance values of the first half plus the first prediction of the second half is used as input by the model to generate the second prediction.
3. Finally, the *N*th prediction uses the previous *N* − 1 predictions. If N−1<30, the remaining inputs are taken from the final high-speed values in the first half.

At the start of the second half, the number of player events for the half is unknown, and for the purpose of the experiment, we assume this number to be equal to the number of events in the first half. Thus, the recursive prediction process is repeated as many times as the number of events performed by the player during the first half.

It was decided to use customised models per player (as opposed to one model for all player positions) by training and testing players separately. The players involved are those who played during the first half of the tested game and played in at least one of the previous games. The set of features used is the same for each model. Three separate models were applied to solve the predictive task:

1. XGBoost [37] with the following hyperparameters: *n_estimators*, 100; *learning_rate*, 0.1; *max_depth*, 3; *min_child_weight*, 1; *colsample_bytree*, 1; *subsample*, 1; *reg_alpha*, 0; *reg_lambda*, 1.
2. A Long Short-Term Memory Network (LSTM) formed by the following layers: Bidirectional LSTM (unites = 64, activation = ‘tanh’) [38], Bidirectional LSTM (unites = 32, activation = ‘tanh’), Dropout (0.20), LSTM (unites = 16, activation = ‘tanh’), Feed Forward (units = 20, activation = ‘relu’), and Feed Forward (units = 1, activation = ‘linear’). The optimiser used is ‘Adam’ [39]; the learning rate and the number of epochs are 0.001 and 50, respectively.
3. A Convolutional Neural Network (CNN) formed by the following layers: one-dimensional convolutional (filters = 64, kernel_size = 5, activation = ‘relu’) [40], one-dimensional convolutional (filters = 32, kernel_size = 3, activation = ‘relu’), one-dimensional convolutional (filters = 10, kernel_size = 5, activation = ‘relu’), one-dimensional MaxPooling (pool_size = 2) [41], Flatten, Feed Forward (40, activation = ‘relu’), Dropout (0.2), and Feed Forward (1, activation = ‘linear’). The optimiser used is ‘Adam’; the learning rate and the number of epochs are equal to 0.001 and 50, respectively.

The LSTM and CNN networks have been implemented in Keras [42]. The predicted event-by-event high-speed distances are summed for each player. In this way, it is possible to obtain the total predicted high-speed distance for the second half. Table 14 compares the predicted value with the true distances covered at high speed for each player. Only those players who played for the entire first and second halves are included. The goalkeeper has been removed from the analysis.

The mean absolute error (MAE) measures the average absolute difference between the predicted values and the actual target values and was used as it is less sensitive to outliers than the root mean squared error. For each model, the MAE between the predicted and the true high-speed distances, displayed in Table 14, has been computed. The MAE is equal to 141.7, 86.2, and 116.4 m for the XGBoost, LSTM, and CNN models, respectively. In short, the LSTM model, to predict the total distance at high speed covered during the second half of the tested game, makes, on average, an error of 86.2 m per player. The average distance at high speed covered by the 10 analysed players during the second half of the game was 358.4 m. On average, the LSTM model makes a percentage error of 24.1% on the total real high-speed distance.

The calculation of the MAE for each event is made impossible by the assumption of an equal number of events between the first and second halves. Indeed, the number of events in the second half (y test) is different from the number of events in the first half (y pred) (Table 14).

We should make clear the limitations of this particular case study. The high-speed distance in the second half could be estimated by fitness coaches by combining the average speed and the peaks of the speed of players. However, our goal is to demonstrate the possibilities using this novel feature set. As this is the first attempt to combine GPS data and machine learning models to predict future high-speed distances, it represents an initial baseline for future comparisons in this task.

## 6. Conclusions

The application of wearable sensor data to analyse the movements, actions, and positions of players for decision support is underused in terms of the potential exploitation of data. The existing literature describes relatively conservative approaches in how wearable sensor data are processed and applied. To the best of the authors’ knowledge, this is the first framework-based approach to process raw GPS data into a feature set suitable for supervised and unsupervised machine learning tasks in the sporting domain. The overall process comprises a number of distinct steps. A temporal rollup to 1 s values is performed by computing the centroid of the locations and the average speed in the 10 observations per second. The average speed is subsequently converted to action labels, which are well understood in the literature. Consecutive rows showing the same actions are aggregated, and several features are computed to form the Actions dataset. The DetectEvent algorithm uses the Actions dataset to group actions into a single event by collecting summary features, to build the Events dataset.

The Actions dataset was validated by comparison with metrics presented in the related literature. A machine learning case study was developed using the Events dataset to show one potential application of the feature engineering methodology. The problem of predicting the high-speed distance covered by players in the second half of a game based on past games and the first half is a new idea, and while this represents a relatively simple study, it represents a baseline for future improvements.

It is important to highlight that this work provides a relatively small case study demonstrating the effectiveness and comparative capabilities of machine learning models. Future research is deliberately planned to enhance the selection, training, and evaluation of machine learning models to ensure the accuracy and stability of the models. Using the dataset created by the work presented here, we are currently focusing on graph-based machine learning for predicting high-speed and distance events late in games; ensemble learning models to predict the future sequence of actions; and anomaly detection functions to detect unusual event sequences within games.

## Figures and Tables

**Table 1:** Summary table presenting the main contributions of related research.

Authors	Year	Data	Contribution
Etemad et al. [21]	2018	GeoLife GPS dataset [33]	Framework based on feature extraction, noise removal, and Random Forest to predict transportation modes from GPS trajectories
Wang et al. [21]	2018	GeoLife GPS dataset [33]	Transportation mode classification method based on feature engineering and Light Gradient Boosting Machine
Zheng et al. [24]	2018	20,349 trajectories produced by 103 locomotives, where each trajectory is composed of a sequence of latitude and longitude coordinates	Methods for extracting driving segmented standard deviation features combined with classical features to improve modes of driving railway train classification performances
Guo et al. [22]	2020	GeoLife GPS dataset [33]	A deep forest and trajectory global feature-based model using only raw GPS data to recognise the transportation modes
Malone et al. [15]	2016	50 male GF players during 30 competitive games using a 4 Hz GPS sensor	Match running profiles of GF players
Malone et al. [28]	2016	50 male GF players during 35 competitive games using a 4 Hz GPS sensor	Comparison of the metabolic power demands between positional groups and examination of the temporal profile of elite GF match play
Cullen et al. [29]	2017	85 male U-18 GF players during 17 competitive games using a 10 Hz GPS sensor	Evaluation of the physiological profiles and activity patterns in club- and county-level under-18 GF players relative to playing position
Ryan et al. [30]	2018	36 male GF players during 19 competitive games using a 4 Hz GPS sensor	Acceleration profile of elite GF match play
Gamble et al. [27]	2019	36 male GF players during five games using a 10 Hz GPS sensor	Running profiles of GF players for playing position and evaluation of the trend of physical performance during the games
Malone et al. [26]	2022	35 male GF players during 32 competitive games	Position- and duration-specific running performance of GF players
Sweeting et al. [31]	2017	12 elite international-level female netball during four competitive games using a Wireless Ad Hoc System for Positioning (WASP)	Method to discover the frequently recurring movement sequences across playing positions during matches using radio frequency data
White et al. [32]	2022	13 professional male rugby players during one Super League match using a 10 Hz GPS sensor	Framework to identify sequential movement sequences using GPS-derived spatiotemporal data in team sports

**Table 2:** Speed zones and parameters.

Speed (m/s)	Speed Zone	Speed Zone ID
$x_{i}\leq$ 0.194	Standing	1
0.194 $<x_{i}\leq$ 2	Walking	2
2 $<x_{i}\leq$ 4	Jogging	3
4 $<x_{i}\leq$ 5.5	Running	4
5.5 $<x_{i}\leq$ 7	High-intensity running	5
$x_{i}>$ 7	Sprinting	6

**Table 3:** Sample of raw data recorded by STATSports Apex 10 Hz sensor. The data shown have been made by the authors to resemble the original data. The names of some columns in the header have been shortened: latitude (Lat.) and longitude (Lon.).

Player ID	Time	Lat.	Lon.	Speed (m/s)
152	15:49:51.5	54.62311	−7.23798	5.60
152	15:49:51.6	54.99321	−7.25665	5.24
152	15:49:51.7	54.85332	−7.23923	5.01
152	15:49:51.8	54.61390	−7.29733	5.28

**Table 4:** Sample of the aggregated data. The names of some columns in the header have been shortened: latitude (Lat.) and longitude (Lon.).

Player ID	Time	Lat.	Lon.	Speed (m/s)
152	15:49:51	54.11398	−7.21719	5.36
152	15:49:52	54.99321	−7.49778	5.60
152	15:49:53	54.11334	−7.89912	5.14
152	15:49:54	54.34509	−7.21196	5.11

**Table 5:** Sample of the aggregated data with labelled actions. The names of some columns in the header have been shortened: latitude (Lat.) and longitude (Lon.).

Player ID	Time	Lat.	Lon.	Speed (m/s)	Action	Acceleration (m^2^/s)
152	15:52:11	54.12311	−7.35743	5.42	4	-
152	15:52:12	54.11387	−7.19723	3.97	3	−1.45
152	15:52:13	54.23334	−7.29989	5.10	4	1.12
152	15:52:14	54.21387	−7.13780	5.07	4	−0.03
152	15:52:15	54.11390	−7.48109	4.99	4	−0.08

**Table 6:** Sample of the aggregated data after the compression of similar actions. The names of some columns in the header have been shortened: start latitude (Start Lat.), start longitude (Start Lon.), end latitude (End Lat.), and end longitude (End Lon.).

Player ID	Start Time	End Time	Start Lat.	Start Lon.	End Lat.	End Lon.	Speed (m/s)	Action	Duration
152	15:53:51	15:53:55	54.123	−7.357	54.224	−7.351	5.36	4	4
152	15:53:56	15:53:58	54.224	−7.351	54.011	−7.391	3.97	3	2
152	15:53:59	15:54:04	54.011	−7.391	54.349	−7.650	4.98	4	5
152	15:54:05	15:54:11	54.349	−7.650	54.012	−7.655	3.83	3	6
152	15:54:12	15:54:14	54.012	−7.655	54.020	−7.657	4.51	4	4

**Table 7:** Direction of movement expressed as labelled turning angles.

Turning Angle (Degrees)	Direction	Direction ID
45 $\leq \tau_{i}$ or $\tau_{i}\geq 315$	Forward	1
$\tau_{i}>$ 45 and $\tau_{i}<135$	Left	2
$\tau_{i}\geq$ 225 and $\tau_{i}<315$	Right	3
$\tau_{i}\geq$ 135 and $\tau_{i}<225$	Backward	4

**Table 8:** Sample of the Actions dataset after events detection. The names of some columns in the header have been shortened: player ID (P.ID), start second (Start Sec.), end second (End Sec.), start latitude (Start Lat.), start longitude (Start Lon.), end latitude (End Lat.), end longitude (End Lon.), direction (Dir.), duration (Dur.), distance (Dist.), and event ID (E.ID).

P.ID	Start Sec.	End Sec.	Start Lat.	Start Lon.	End Lat.	End Lon.	Speed	Action	Dir.	Dur.	Dist.	E.ID
152	54	56	54.55	−7.35	54.11	−7.35	5.36	4	1	4	20.3	31
152	56	57	54.11	−7.35	54.13	−7.36	6.60	5	1	1	6.6	31
152	57	62	54.13	−7.36	54.33	−7.37	4.98	4	3	4	17	31
152	62	71	54.33	−7.37	54.52	−7.34	3.83	2	2	9	18.8	32
152	71	76	54.51	−7.34	54.55	−7.33	4.51	3	4	6	24.5	32

**Table 9:** Schema of the Events dataset at the end of the multistep framework. The names of some columns in the header have been shortened: player ID (P.ID), event ID (E.ID), start second (Start Sec.), end second (End Sec.), avg speed (Avg Sp.), std speed (Std Sp.), duration (Dur.), standard deviation duration (Std Dur.), distance (Dis.), high-speed distance (High-Speed Dist.), and unique turning angle (U.T.A.).

P.ID	E.ID	Start Sec.	End Sec.	Start Lat	Start Lon	End Lat	End Lon	Avg Sp.	Std Sp.	Dur.	Std Dur.	Dis.	High-Speed Dist.	U. T. A.
152	4	23	27	54.55	−7.35	54.11	−7.35	5.3	0.3	4	0	23.5	3.1	2
152	5	27	29	54.51	−7.31	54.11	−7.35	3.2	0.1	2	0	5.9	0	1
152	6	29	39	54.21	−7.31	54.11	−7.31	5.7	1.7	10	2.6	60	6.7	3
152	7	29	43	54.01	−7.01	54.18	−7.39	3.3	1.2	14	3.4	36.9	0	4
152	8	43	48	54.11	−7.42	54.16	−7.30	6.3	0.5	5	1.9	41.2	11.1	1

**Table 10:** Half by half analysis: action count, average, and maximum duration, sorted by count of actions.

Game ID	Half	Count	Duration Avg (s)	Duration Std (s)	Duration Max (s)
893	1	4536	2.96	2.57	23
997	1	4308	2.88	2.59	22
788	1	4193	2.80	2.36	25
973	1	4165	2.83	2.47	26
869	2	4129	2.80	2.36	23
893	2	4083	2.89	2.53	26
811	1	4071	2.97	2.64	25
946	1	4018	2.81	2.41	23
889	1	3984	2.94	2.60	25
873	1	3983	2.83	2.48	24
997	2	3972	2.98	2.61	25
934	1	3919	2.95	2.59	23
811	2	3844	2.99	2.63	37
869	1	3806	2.79	2.34	25
788	2	3729	2.85	2.46	25
873	2	3677	2.91	2.49	23
838	1	3662	2.87	2.53	24
973	2	3614	2.85	2.45	25
946	2	3594	2.74	2.35	26
934	2	3502	2.89	2.50	26
889	2	3441	2.89	2.48	25
838	2	3417	2.86	2.61	29

**Table 11:** Count, average, and maximum duration and distance covered for each type of action during the sampled games, sorted by average distance.

Action	Game ID	Count	Duration Avg (s)	Duration Max (s)	Distance Avg (m)	Distance Max (m)
Sprint	873	168	2.4	7	17.8	58.8
	973	149	2.3	9	17.1	71.1
	889	153	2.3	7	17.1	57.4
	869	178	2.2	8	16.5	60.8
	788	168	2.2	6	16.4	49.2
	838	131	2.1	8	16.1	62.4
	893	171	2.1	9	16.0	66.6
	997	146	2.0	6	15.5	47.4
	934	162	2.0	7	15.1	53.2
	811	136	2.0	7	15.0	52.5
	946	144	1.9	6	14.7	49.8
High-intensity running	869	911	2.2	11	13.6	68.2
	889	820	2.2	12	13.3	70.8
	997	868	2.2	13	13.2	79.3
	811	784	2.1	11	13.1	71.5
	973	837	2.1	14	13.00	86.8
	893	906	2.1	10	12.8	65.0
	838	726	2.0	10	12.6	63.0
	873	836	2.0	10	12.5	63.0
	946	780	2.0	10	12.4	60.0
	934	781	2.0	10	12.3	64.0
	788	858	2.0	11	12.1	67.1
Running	997	2589	2.3	21	10.9	96.6
	893	2653	2.4	20	10.9	94.0
	811	2417	2.3	24	10.8	112.8
	973	2449	2.3	18	10.8	86.4
	788	2451	2.3	22	10.6	110.0
	869	2468	2.3	20	10.6	96.0
	873	2337	2.3	16	10.6	76.8
	889	2313	2.3	16	10.6	76.8
	934	2256	2.2	16	10.4	76.8
	946	2357	2.2	15	10.3	69.0
	838	2138	2.2	14	10.1	61.6
Jogging	811	4578	3.5	37	10.5	107.3
	934	4222	3.5	26	10.4	72.8
	893	4889	3.4	26	10.3	85.8
	889	4139	3.4	25	10.3	82.5
	997	4677	3.4	25	10.3	77.5
	838	4084	3.4	29	10.1	84.1
	873	4319	3.3	24	10.0	74.4
	973	4344	3.3	26	9.9	80.6
	788	4445	3.3	25	9.8	80.0
	946	4331	3.2	26	9.7	84.0
	869	4378	3.2	25	9.6	70.0

**Table 12:** Count of events, actions, average number and duration of actions in an event, and maximum number and duration of actions in an event per half of the investigated games, sorted by count of events.

Game ID	Half	Events Count	Duration Avg (s)	Duration Max (s)	Distance Avg (m)	Distance Max (m)	H. S. Dist. Avg (m)	H. S. Dist. Max (m)
893	1	1947	7.0	48	25.4	189.5	3.3	117.4
997	1	1756	7.2	52	26.8	236.8	4.3	87.0
811	1	1708	7.2	63	26.4	216.7	3.7	86.5
869	2	1676	7.0	38	26.5	158.9	5.1	97.7
946	1	1668	6.9	38	25.2	148.2	3.8	86.9
973	1	1661	7.2	45	26.7	198.8	4.0	98.1
788	1	1654	7.3	44	27.0	199.0	4.1	89.0
997	2	1645	7.3	48	26.9	191.4	3.7	108.8
811	2	1637	7.2	52	26.0	190.4	3.6	80.2
889	1	1631	7.3	40	26.8	182.9	4.4	99.9
893	2	1604	7.5	54	28.1	214.7	4.8	113.5
788	2	1594	6.8	52	24.9	236.9	4.0	84.8
873	1	1590	7.2	38	26.8	182.7	4.6	82.5
869	1	1575	6.9	49	25.6	188.8	4.4	112.9
838	1	1568	6.8	48	24.8	188.9	3.5	82.4
973	2	1567	6.7	48	25.3	226.2	4.3	108.6
873	2	1559	7.0	49	25.7	190.2	3.8	82.5
934	1	1554	7.5	49	27.6	209.3	4.1	92.7
946	2	1482	6.8	45	24.8	179.0	3.7	93.5
838	2	1450	6.8	38	25.1	156.9	3.9	91.5
934	2	1428	7.2	44	26.5	163.5	4.0	70.6
889	2	1369	7.4	72	27.7	264.1	4.9	126.2

**Table 13:** Structure of the transformed dataset used for the prediction of high-speed distance (H.S.D.) covered by players during the second half of a game. The **target feature** to predict is the variable **31st H.S.D** located at the end of the sequence.

P.ID	1st H.S.D.	2nd H.S.D.	3rd H.S.D.	…	…	…	29th H.S.D.	30th H.S.D.	31st H.S.D.
152	0	0	0	…	…	…	3.2	0	5.1
152	0	0	0	…	…	…	0	5.1	0
152	0	0	15.4	…	…	…	5.1	0	0
152	0	15.4	0	…	…	…	0	0	21.3
152	15.4	0	2.6	…	…	…	0	21.3	0

**Table 14:** Player by player predictions and true values for the total distance at high speed during the second half of a randomly tested game using XGboost, LSTM, and CNN models defined in Section 5. The values presented in the tables are expressed in metres.

Player ID	Pred. H.S.D. XGBoost	Pred. H.S.D. LSTM	Pred. H.S.D. CNN	Actual H.S.D.	True Number of Events	Number of Events Used for Pred.
146	381.9	528.3	344.0	543.6	211	196
152	633.3	422.3	878.5	367.5	180	176
163	642.8	432.1	281.9	285.3	76	126
194	474.5	368.0	249.8	281.8	116	150
201	240.9	340.5	370.2	341.9	199	193
220	336.7	590.1	378.2	341.9	234	286
229	239.5	422.7	355.2	292.8	265	282
236	498.4	335.9	252.3	457.0	234	244
244	519.0	446.1	340.8	405.8	189	222
278	141.3	284.9	288.2	266.6	180	175

## Data Availability

Dataset available on request from the authors.

## Abbreviations

The following abbreviations are used in this manuscript:

GPS	Global Positioning System
IFTS	Invasion Field-Based Team Sport
GF	Gaelic Football
ML	Machine Learning
